# Evaluation of multiple generative large language models on neurology board-style questions

**DOI:** 10.3389/fdgth.2025.1737882

**Published:** 2026-01-05

**Authors:** Mohammad Almomani, Vijaya Valaparla, James Weatherhead, Xiang Fang, Alok Dabi, Chih-Ying Li, Peter McCaffrey, Dan Hier, Jorge Mario Rodríguez-Fernández

**Affiliations:** 1 Department of Neurology, University of Texas Medical Branch (UTMB), Galveston, TX, United States; 2 School of Biomedical Sciences, University of Texas Medical Branch (UTMB), Galveston, TX, United States; 3 Department of Occupational Therapy, University of Texas Medical Branch (UTMB), Galveston, TX, United States; 4 Department of Pathology, University of Texas Medical Branch (UTMB), Galveston, TX, United States; 5 Department of Neurology and Rehabilitation, University of Illinois at Chicago, Chicago, IL, United States; 6 Department of Electrical and Computer Engineering, Missouri University of Science and Technology, Rolla, MO, United States

**Keywords:** artificial intelligence, large language models, neurology education, board examinations, model performance analysis

## Abstract

**Objective:**

To compare the performance of eight large language models (LLMs) with neurology residents on board-style multiple-choice questions across seven subspecialties and two cognitive levels.

**Methods:**

In a cross-sectional benchmarking study, we evaluated Bard, Claude, Gemini v1, Gemini 2.5, ChatGPT-3.5, ChatGPT-4, ChatGPT-4o, and ChatGPT-5 using 107 text-only items spanning movement disorders, vascular neurology, neuroanatomy, neuroimmunology, epilepsy, neuromuscular disease, and neuro-infectious disease. Items were labeled as lower- or higher-order per Bloom's taxonomy by two neurologists. Models answered each item in a fresh session and reported confidence and Bloom classification. Residents completed the same set under exam-like conditions. Outcomes included overall and domain accuracies, guessing-adjusted accuracy, confidence–accuracy calibration (Spearman *ρ*), agreement with expert Bloom labels (Cohen *κ*), and inter-generation scaling (linear regression of topic-level accuracies). Group differences used Fisher exact or *χ*^2^ tests with Bonferroni correction.

**Results:**

Residents scored 64.9%. ChatGPT-5 achieved 84.1% and ChatGPT-4o 81.3%, followed by Gemini 2.5 at 77.6% and ChatGPT-4 at 68.2%; Claude (56.1%), Bard (54.2%), ChatGPT-3.5 (53.3%), and Gemini v1 (39.3%) underperformed residents. On higher-order items, ChatGPT-5 (86%) and ChatGPT-4o (82.5%) maintained superiority; Gemini 2.5 matched 82.5%. Guessing-adjusted accuracy preserved rank order (ChatGPT-5 78.8%, ChatGPT-4o 75.1%, Gemini 2.5 70.1%). Confidence–accuracy calibration was weak across models. Inter-generation scaling was strong within the ChatGPT lineage (ChatGPT-4 to 4o *R*^2^ = 0.765, *p* = 0.010; 4o to 5 *R*^2^ = 0.908, *p* < 0.001) but absent for Gemini v1 to 2.5 (R^2^ = 0.002, *p* = 0.918), suggesting discontinuous improvements.

**Conclusions:**

LLMs—particularly ChatGPT-5 and ChatGPT-4o—exceeded resident performance on text-based neurology board-style questions across subspecialties and cognitive levels. Gemini 2.5 showed substantial gains over v1 but with domain-uneven scaling. Given weak confidence calibration, LLMs should be integrated as supervised educational adjuncts with ongoing validation, version governance, and transparent metadata to support safe use in neurology education.

## Introduction

Rapid advancements in artificial intelligence (AI) have transformed many aspects of medicine, with generative AI emerging as a particularly promising innovation in neurology (1). Large language models (LLMs), a subset of AI, hold potential for enhancing diagnostic accuracy, advancing therapeutics, and contributing to patient and clinician education (2). Machine learning approaches have already demonstrated applicability across neurology subspecialties by improving the analysis of complex clinical data and supporting individualized outcome prediction (3).

LLMs are trained on vast corpora using deep learning and have exhibited strong performance in natural language processing tasks such as summarization, translation, and question answering. Recently, models such as OpenAI's ChatGPT and Microsoft's Bing Chat have been evaluated on standardized medical licensing examinations, including the United States Medical Licensing Examination (USMLE), where they achieved near-pass or passing scores (4, 5). However, their application in neurology remains relatively limited. One study reported that ChatGPT-3.5 performed below the average passing threshold on neurosurgery board examinations, whereas ChatGPT-4 exceeded passing standards (6).

Neurology board examinations present unique challenges, requiring mastery of complex clinical narratives integrating neuroanatomy, neuropathology, and neurophysiology. These assessments demand higher-order reasoning, nuanced differential diagnosis, and synthesis of multifaceted case presentations (7). While prior research has examined LLMs on general medical board-style assessments, their performance in neurology-specific examinations has not been systematically characterized across multiple model generations (8–10). This study addresses that gap by assessing the accuracy, reasoning capabilities, and limitations of LLMs in neurology board–style examinations and by comparing their performance with neurology residents. By understanding both the strengths and weaknesses of LLMs in this specialized context, we aim to evaluate their role in medical education and clinical decision support, paving the way for future AI-assisted advancements in neurology.

## Methods

### Study design and ethical considerations

This was a cross-sectional exploratory study. Institutional review board (IRB) exemption was obtained because the project did not involve human participants or identifiable patient information and was conducted as an educational benchmarking activity using deidentified data.

### Question development and classification

Multiple-choice questions were written and reviewed by board-certified physicians to resemble neurology board examinations. All questions were written *de novo* by board-certified neurologists and were not adapted or copied from commercial question banks or public online sources. All items were text-based, excluded radiologic or pathologic images, and followed a single-best-answer format. A total of 107 questions were developed and categorized by subspecialty: epilepsy (*n* = 8), movement disorders (*n* = 13), neuroanatomy (*n* = 25), neuroimmunology (*n* = 14), neuroinfectious disease (*n* = 17), neuromuscular disease (*n* = 17), and vascular neurology (*n* = 13).

Each question was further classified according to Bloom's taxonomy as lower-order (remembering or basic understanding) or higher-order (application, analysis, or evaluation). Classification was performed independently by two board-certified physicians (V.V., J.M.R.F.), with disagreements resolved by consensus (11). Each large language model (LLM) was then asked to classify each item as higher- or lower-order and to provide confidence ratings for both classification and answer selection. The following standardized prompt was used (verbatim):

You are a medical doctor and are taking the neurology board exam. The board exam consists of multiple choice questions. All output that you give must be in CSV format with the following six columns (1) Question number (2) Return the answer letter (3) Give an explanation (4) Rate your own confidence in your answer based on a Likert scale that has the following grades: 1 = no confidence [stating it does not know]; 2 = little confidence [i.e., maybe]; 3 = some confidence; 4 = confidence [i.e., likely]; 5 = high confidence [stating answer and explanation without doubt] (5) Classify the question into the following two categories: (1) lower order questions that probe remembering and basic understanding, and (2) higher order question where knowledge needs to be applied, analysis capabilities are examined, or evaluation is needed (return “Higher” or “Lower”) (6). Rate the confidence of your classification into these categories based on the Likert scale that has the following grades1 = no confidence [stating it does not know]; 2 = little confidence [i.e., maybe]; 3 = some confidence; 4 = confidence [ie, likely]; 5 = high confidence [stating answer and explanation without doubt]) Your output must look like the following row header: {“questionnumber”:…,“answerletter”:…,“reasoning”:…,“confidence_answer_likert“:…,”classification“:…,” confidence_classification_likert“:…”}.

Although the prompt includes an exam-level framing, each question was entered into a separate, isolated chat session to maintain strict question-level independence and prevent conversational carry-over. This phrasing was retained because it reliably improved adherence to the required CSV output format without creating shared multi-question context.

### Model evaluation procedures

All models were evaluated through their publicly available, web-based graphical user interfaces (GUIs) to simulate real-world clinician and trainee use. Each question was entered in a new, independent chat session to prevent conversational context from influencing subsequent responses. The interaction date for each model was recorded to document the version tested. To preserve real-world usability and reflect typical clinician interactions with public LLM interfaces, model responses were collected directly from the web platforms without automated post-processing or formatting scripts. When the model's output did not fully adhere to the required CSV structure, the prompt was re-submitted once in a new, isolated session to obtain a complete response. No question was ever regenerated after the model had visibility of the correct answer, and answer content was not manually modified.

### Resident comparison group

Neurology residents completed the same 107 board-style multiple-choice questions covering all major subspecialties. All results were deidentified before analysis to maintain confidentiality. A total of 16 neurology residents participated (PGY-4 *n* = 4, PGY-3 *n* = 6, PGY-2 *n* = 6), and participation was voluntary as part of an educational benchmarking activity. The study adhered to institutional standards for ethical conduct and data privacy. Question content and difficulty were designed to reflect those of the American Academy of Neurology (AAN) Residency In-Service Training Examination and the American Board of Psychiatry and Neurology (ABPN) Certification Examination (12, 13).

Eight LLMs were tested using their publicly available web-based graphical user interfaces to replicate real-world conditions. Each question was entered into a new, independent session to prevent conversational carry-over. The models included Bard, Claude-1, Gemini v1, Gemini 2.5, ChatGPT-3.5, ChatGPT-4, ChatGPT-4o, and ChatGPT-5. All models were given identical prompts instructing them to select the most appropriate answer, provide a short explanation, classify the item as higher- or lower-order according to Bloom's taxonomy, and rate their confidence for both the answer and classification on a five-point Likert scale (1 = no confidence to 5 = high confidence). Each prompt was formatted for CSV output to facilitate later analysis, and all sessions were time-stamped to record the model version tested. These models were selected because they were publicly accessible, widely used by clinicians and trainees, and representative of major contemporary LLM families. Other contemporary or domain-specific models that were not publicly accessible through stable consumer-facing interfaces at the time of testing were not included in this study.

For human comparison, neurology residents at the University of Texas Medical Branch completed the same 107 questions under controlled exam conditions. All scores were deidentified before analysis to ensure anonymity and compliance with institutional privacy standards.

### Outcome measures and statistical analysis

Accuracy was calculated as the proportion of correctly answered questions for each model and for the resident cohort. Comparisons between model and resident performance were performed using Fisher's exact test with Bonferroni correction for multiple comparisons. Accuracy differences between higher- and lower-order questions were evaluated using *χ*^2^ tests, and corrected accuracy was computed using the formula: number correct—[number incorrect ÷ (k—1)], where *k* represents the number of answer options. Associations between model confidence and correctness were assessed with the Mann–Whitney *U* test and Spearman rank correlation (*ρ*), while agreement between model classifications and expert labels was measured with Cohen's *κ*. Longitudinal model-to-model improvement was examined through linear regression of subspecialty-specific accuracies, focusing on the transitions ChatGPT-4 and ChatGPT-4o, ChatGPT-4o and ChatGPT-5, and Gemini v1 and Gemini 2.5, with regression coefficients (*β*₁), coefficients of determination (*R*^2^), and *p* values reported. All statistical analyses were conducted using R (version 4.0.5; R Foundation for Statistical Computing, Vienna, Austria), and statistical significance was defined as two-tailed *p* < .05.

## Results

### Overall model and resident performance

A total of 107 items were analyzed across seven subspecialties. Neurology residents achieved a mean overall accuracy of 64.9% across all 107 questions. Among the eight large language models evaluated, ChatGPT-5 demonstrated the highest performance, achieving 84.1% accuracy, followed by ChatGPT-4o at 81.3% and Gemini 2.5 at 77.6%. Each of these models significantly outperformed the resident cohort. ChatGPT-4 scored 68.2%, only modestly above resident performance, whereas Claude (56.1%), Bard (54.2%), and ChatGPT-3.5 (53.3%) clustered below the resident mean. Gemini v1 was the weakest model, with an overall accuracy of 39.3%. Pairwise Fisher's exact testing confirmed clear separation between higher- and lower-performing model tiers: ChatGPT-5 and ChatGPT-4o differed significantly from nearly all other models (*p* < 0.001) but not from each other (*p* = 0.718). ChatGPT-4 was significantly stronger than Gemini v1 (*p* < 0.001) but statistically similar to Gemini 2.5 (*p* = 0.166). At the lower end, Bard and ChatGPT-3.5 were indistinguishable (*p* = 1.0), as were Bard and Claude (*p* = 0.891). Gemini v1 was decisively outperformed by Gemini 2.5 (*p* < 0.001), confirming a large inter-version improvement.

Detailed accuracy by subspecialty and question category is presented in Table 1, with corresponding pairwise statistical comparisons shown in Table 2 and calibration metrics summarized in Table 3.

**Table 1:** Overall and domain-specific accuracy, question category, and corrected accuracy.

Topic	Questions	Students (%)	Bard (%)	Claude (%)	Gemini v1 (%)	Gemini 2.5 (%)	ChatGPT-3.5 (%)	ChatGPT-4 (%)	ChatGPT-4o (%)	ChatGPT-5 (%)
Overall	107	64.9	54.2	56.1	39.3	77.6	53.3	68.2	81.3	84.1
Movement disorders	13	72.6	38.5	53.9	38.5	76.9	38.5	53.9	76.9	76.9
Neuroanatomy	25	70.3	44	48	40	96	44	80	88	96
Neuro-infections	17	69.3	47.1	52.9	41.2	76.5	64.7	64.7	70.6	70.6
Neuroimmunology	14	66.1	78.6	78.6	35.7	64.3	78.6	100	100	100
Epilepsy	8	65.6	62.5	62.5	50	62.5	62.5	62.5	75	75
Vascular neurology	13	58.1	84.6	61.5	46.2	76.9	53.9	61.5	76.9	76.9
Neuromuscular	17	50.4	41.2	47.1	29.4	70.6	41.2	47.1	76.5	82.4
Lower-order	50	66.4	56	66	42	72	60	68	80	82
Higher-order	57	63.5	52.6	47.4	36.8	82.5	47.4	68.4	82.5	86
Corrected accuracy	–	–	38.9	41.4	19	70.1	37.7	57.6	75.1	78.8

**Table 2:** Pairwise fisher exact test *p*-values.

Model 1	Model 2	*p*-value
ChatGPT-3.5	ChatGPT-4	0.036
ChatGPT-3.5	ChatGPT-4o	1.95 × 10^−⁵^
ChatGPT-3.5	ChatGPT-5	1.69 × 10^−^⁶
ChatGPT-3.5	Bard	1
ChatGPT-3.5	Claude	0.784
ChatGPT-3.5	Gemini v1	0.055
ChatGPT-3.5	Gemini 2.5	2.97 × 10^−^⁴
ChatGPT-4	ChatGPT-4o	0.04
ChatGPT-4	ChatGPT-5	0.0098
ChatGPT-4	Bard	0.049
ChatGPT-4	Claude	0.091
ChatGPT-4	Gemini v1	3.48 × 10^−^⁵
ChatGPT-4	Gemini 2.5	0.166
ChatGPT-4o	ChatGPT-5	0.718
ChatGPT-4o	Bard	3.52 × 10^−^⁵
ChatGPT-4o	Claude	1.10 × 10^−^⁴
ChatGPT-4o	Gemini v1	3.83 × 10^−1^⁰
ChatGPT-4o	Gemini 2.5	0.612
ChatGPT-5	Bard	3.25 × 10^−^⁶
ChatGPT-5	Claude	1.15 × 10^−^⁵
ChatGPT-5	Gemini v1	1.37 × 10^−11^
ChatGPT-5	Gemini 2.5	0.297
Bard	Claude	0.891
Bard	Gemini v1	0.0396
Bard	Gemini 2.5	4.96 × 10^−^⁴
Claude	Gemini v1	0.0198
Claude	Gemini 2.5	1.32 × 10^−3^
Gemini v1	Gemini 2.5	1.86 × 10^−^⁸

**Table 3:** Calibration and agreement per model.

Model	Cohen's *κ*	Spearman *ρ*	*p*-value
Bard	0.078	0.053	0.588
Claude	0.03	−0.033	0.739
Gemini v1	0.009	0.184	0.058
Gemini 2.5	0.237	−0.074	0.447
ChatGPT-3.5	0.021	−0.068	0.487
ChatGPT-4	0.085	0.176	0.07
ChatGPT-4o	0.218	−0.081	0.404
ChatGPT-5	0.217	−0.090	0.4

### Performance by cognitive level

When questions were stratified by cognitive complexity, performance gradients became evident. On lower-order items, residents achieved 66.4%, while ChatGPT-5 (82%), ChatGPT-4o (80%), and Gemini 2.5 (72%) maintained clear advantages. ChatGPT-4 (68%) performed comparably to residents, whereas Claude (66%), Bard (56%), and ChatGPT-3.5 (60%) offered minimal improvement. Gemini v1 again performed worst (42%). For higher-order questions, resident accuracy declined slightly to 63.5%, while the gap between models widened: ChatGPT-5 (86%) and ChatGPT-4o (82.5%) led all models, Gemini 2.5 matched them at 82.5%, and ChatGPT-4 held steady at 68.4%. Claude (47.4%), Bard (52.6%), ChatGPT-3.5 (47.4%), and Gemini v1 (36.8%) demonstrated marked deterioration on higher-order reasoning tasks.

### Subspecialty-level accuracy

Subspecialty analysis revealed similar hierarchical trends. In movement disorders, residents attained 72.6% accuracy, outperforming lower-tier models but equaled by ChatGPT-5, ChatGPT-4o, and Gemini 2.5 (all 76.9%). In neuroanatomy, advanced models excelled, with ChatGPT-5 and Gemini 2.5 each reaching 96%, ChatGPT-4o at 88%, and ChatGPT-4 at 80%, all surpassing residents (70.3%). In neuroimmunology, ChatGPT-5, ChatGPT-4o, and ChatGPT-4 achieved perfect scores (100%), far above residents (66.1%), while Claude and Bard performed similarly (78.6%), and Gemini v1 remained lowest (35.7%). Vascular neurology produced the most unexpected finding: Bard achieved the highest single-domain score (84.6%), exceeding residents (58.1%) and even the strongest models (ChatGPT-4o, ChatGPT-5, and Gemini 2.5, all 76.9%). In neuromuscular disease—the most challenging section for residents (50.4%)—the advanced models again separated clearly, with ChatGPT-5 at 82.4%, ChatGPT-4o at 76.5%, and Gemini 2.5 at 70.6%, all outperforming other models by wide margins. In epilepsy and neuro-infectious disease, model and human accuracies converged more closely, with residents at 65%–69% and top models modestly higher (70%–76%).

### Guessing-adjusted accuracy and calibration

Adjusting for random guessing, corrected accuracy preserved the same performance hierarchy. ChatGPT-5 (78.8%) and ChatGPT-4o (75.1%) remained the strongest models, followed by Gemini 2.5 (70.1%) and ChatGPT-4 (57.6%). The lower-performing group—Claude (41.4%), Bard (38.9%), ChatGPT-3.5 (37.7%), and Gemini v1 (19.0%)—clustered near chance level, indicating limited comprehension.

Confidence-accuracy calibration analyses revealed generally weak and nonsignificant correlations. ChatGPT-4 (*ρ* = 0.176, *p* = 0.070) and Gemini v1 (*ρ* = 0.184, *p* = 0.058) showed mild positive trends, suggesting that higher reported confidence modestly aligned with accuracy. By contrast, ChatGPT-5 (*ρ* = −0.090, *p* = 0.400), ChatGPT-4o (*ρ* = −0.081, *p* = 0.404), and Gemini 2.5 (*ρ* = −0.074, *p* = 0.447) demonstrated weak negative associations, indicating poor self-calibration. Agreement with expert classifications by Bloom's taxonomy also varied: Gemini 2.5 (*κ* = 0.237), ChatGPT-4o (*κ* = 0.218), and ChatGPT-5 (*κ* = 0.217) exhibited the highest concordance with expert labels, whereas ChatGPT-4 (*κ* = 0.085) and Bard (*κ* = 0.078) showed only slight agreement, and Claude (*κ* = 0.030), ChatGPT-3.5 (*κ* = 0.021), and Gemini v1 (*κ* = 0.009) approached random alignment.

### Inter-model improvement across generations

**Figure 1:**
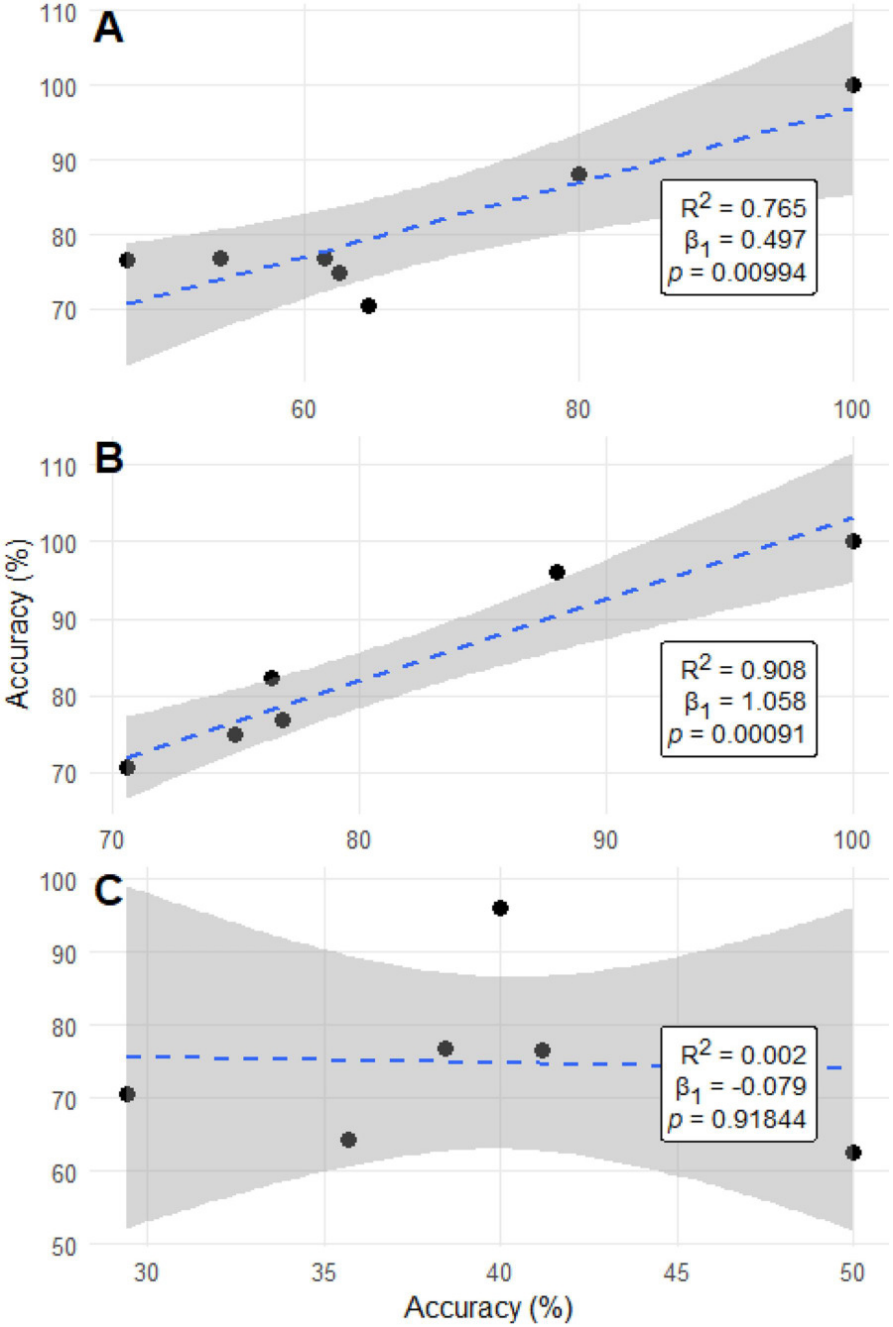
Linear regression analyses comparing accuracy between consecutive large language model generations. Panels **(A–C**) display regression analyses evaluating the relationship between the accuracies of successive large language model versions across neurology question domains. Panel **(A)** compares GPT-4 and GPT-4o; panel **(B)** compares GPT-4o and GPT-5; and panel **(C)** compares Gemini version 1 and Gemini 2.5. Each plot includes the fitted regression line (dashed blue), 95% confidence interval (gray shading), and corresponding model metrics (*R*^2^, *β*, *p*) shown in the lower right corner of each panel. Both axes represent model accuracy expressed as a percentage.

**Figure 2:**
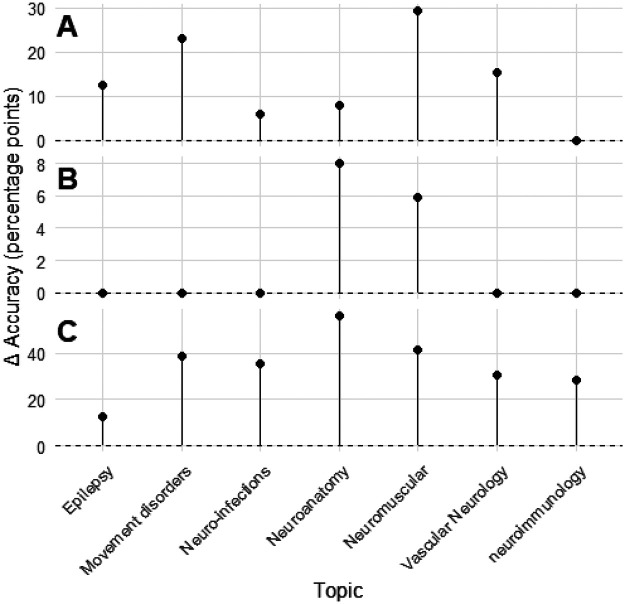
Change in per-topic accuracy between consecutive large language model generations. Panels **(A–C)** depict the per-topic change (Δ) in accuracy, expressed in percentage points, between successive large language model versions. Panel **(A)** compares GPT-4 and GPT-4o, **(B)** compares GPT-4o and GPT-5, and **(C)** compares Gemini version 1 and Gemini 2.5. Each point represents a neurology topic, with vertical lines extending from zero to the observed change in accuracy. Positive values indicate improvement relative to the preceding model.

Regression analyses demonstrated consistent incremental improvement across ChatGPT model generations. Topic-level accuracies for ChatGPT-4 and ChatGPT-4o were strongly correlated (R^2^ = 0.765, *p* = 0.0099), with ChatGPT-4o gaining roughly half a percentage point for each one-point increase in ChatGPT-4 performance (*β* = 0.497, 95% CI 0.18–0.81). The transition from ChatGPT-4o to ChatGPT-5 showed even greater linearity (R^2^ = 0.908, *p* < 0.001), with near-uniform accuracy improvements across subspecialties (*β* = 1.06, 95% CI 0.67–1.45). By contrast, Gemini v1 and Gemini 2.5 demonstrated virtually no linear relationship (*R*^2^ = 0.002, *p* = 0.918), reflecting discontinuous and domain-inconsistent progress. Figure 1 illustrates inter-model regression relationships, while Figure 2 depicts per-topic change in accuracy between large language model generations. These findings indicate that ChatGPT iterations improved predictably and systematically across subspecialties, while Gemini's evolution was abrupt but uneven.

## Discussion

LLMs are beginning to supplement traditional teaching methods in medical education, but their application in neurology requires careful evaluation (4, 5). While their application in neurology remains underexplored, the specialty's reliance on nuanced clinical reasoning, pattern recognition, and hypothesis generation presents both opportunities and challenges for AI integration (1, 2). Evaluating LLMs in neurology-specific examinations is therefore critical to guide their optimal use in training, curriculum design, and board preparation (7, 14).

In this study, eight LLMs were compared against neurology residents across a board-style multiple-choice examination spanning seven subspecialties. Consistent with prior evaluations in neurosurgery (6), radiology (8), and ophthalmology (10), newer models clearly outperformed earlier versions. ChatGPT-5 achieved the highest overall accuracy, followed closely by ChatGPT-4o, both exceeding resident performance across most subspecialties. Gemini 2.5 also demonstrated substantial improvement over Gemini v1, achieving accuracy closer to ChatGPT-4, though with more variability across topics. Bard, Claude, and ChatGPT-3.5 performed below resident levels, aligning with reports of uneven LLM performance across medical disciplines (9). Our findings complement international evidence. In a recent study of practicing neurologists in Spain, Ros-Arlanzón and Pérez-Sempere found that ChatGPT-4 outperformed clinicians on a high-stakes neurology certification exam administered in Spanish, whereas ChatGPT-3.5 performed below the clinician cohort. Their results reinforce the generational performance gradient observed in our analysis (15).

A notable finding was the systematic, domain-consistent improvement observed across ChatGPT generations, contrasting with the discontinuous leap between Gemini v1 and Gemini 2.5. Model improvement analyses demonstrated strong positive correlations between ChatGPT-4 and ChatGPT-4o, and between ChatGPT-4o and ChatGPT-5, indicating steady, distributed gains across neurology subspecialties—results consistent with prior evidence of iterative refinement across successive LLM generations (16, 17). In contrast, Gemini's performance gains were abrupt and uneven; although Gemini 2.5 achieved substantially higher accuracy than v1, these changes were not significantly correlated across domains, raising concerns about the reproducibility and stability of its progress. This difference underscores that evaluating LLMs for neurology education requires attention not only to absolute accuracy but also to the *trajectory* and *predictability* of model advancement. Furthermore, this finding highlights broader implications for version governance. Because up-versioning or down-versioning may not yield predictable changes in performance, maintaining validated, version-specific performance data for each deployed model becomes essential. Transitions between model generations should therefore be deliberate, transparent, and monitored for downstream impact. As model routing and multimodal agent frameworks proliferate—where automatic switching between models or versions may occur dynamically—such oversight grows increasingly complex. Future work and emerging standards must address not only model performance but also the systematic recording of version metadata, its inclusion in audit trails, and the development of mechanisms enabling agentic tools and routers to make model-selection decisions aligned with safe, optimal medical reasoning.

Confidence–accuracy calibration emerged as a persistent limitation. ChatGPT-4o and ChatGPT-5 both showed weak negative correlations between confidence and correctness, while Gemini v1 and Gemini 2.5 demonstrated inconsistent calibration. This pattern mirrors prior evidence that LLMs frequently overestimate correctness by 20%–60%, raising concern for educational or clinical contexts where confidently incorrect outputs may mislead learners (18). In our analysis, corrected accuracy further magnified performance differences: Gemini v1 dropped to near-chance levels, underscoring the risk of relying on raw outputs when confidence misalignment persists.

From an educational standpoint, high-performing models such as ChatGPT-4o, ChatGPT-5, and Gemini 2.5 may serve as effective adjuncts for board preparation, question generation, and literature review (2, 7). Their strengths in factual recall and content summarization make them promising tools for structured learning, yet their limitations in higher-order reasoning highlight the continued need for expert oversight (1). These findings echo broader concerns regarding reproducibility and reliability across prompts (14).

This study has several limitations. The relatively small number of questions within certain subspecialties may have reduced statistical power. We did not assess intra-model variability, hallucination frequency, or multimodal reasoning, each of which constrains the applicability of LLMs in clinical contexts (19, 20). Because LLMs evolve rapidly, the performance reported here may not reflect future iterations (14, 16). Models were tested through publicly available graphical user interfaces, preventing adjustment of inference parameters (e.g., temperature) and limiting reproducibility, though this approach mirrors real-world use (21). These findings apply only to text-based, single-best-answer questions without imaging, waveform, or multimedia elements, which limits generalizability to the multimodal assessments used in neurology (22). Furthermore, the “best-answer” structure lacked a “none of the above” option, which may obscure key failure modes recently identified in LLM testing (23). The prompts themselves combined multiple requests—question response, confidence rating, explanation, Bloom's classification, and CSV formatting—creating a one-shot configuration that could increase variability compared with explicit chain-of-thought prompting. Finally, some board-style questions may overlap with publicly available educational material incorporated into model training data; paradoxically, even seemingly esoteric items can appear online when they reference pathognomonic findings or “classic” examination vignettes.

## Conclusions

LLMs—particularly ChatGPT-4o, ChatGPT-5, and Gemini 2.5—demonstrate strong potential as educational tools in neurology, often matching or exceeding resident performance in subspecialty assessments. However, inconsistent calibration of confidence and limitations in higher-order reasoning restrict their readiness for unsupervised educational or clinical use. Deployment in medical training should be cautious, with robust oversight, accuracy validation, and transparency. As models evolve and agentic AI capabilities mature, their role in neurology education is likely to expand, but careful integration with traditional teaching and expert guidance will remain essential.

## Data Availability

Deidentified model outputs and aggregated question-level accuracy data are available from the corresponding author upon reasonable request for educational replication. No protected health information is included.

## References

[1] Hillis JM Bizzo BC. Use of artificial intelligence in clinical neurology. Semin Neurol. (2022) 42(1):39–47. 10.1055/s-0041-1742180 35576929

[2] Romano MF Shih LC Paschalidis IC Au R Kolachalama VB. Large language models in neurology research and future practice. Neurology. (2023) 101(23):1058–67. 10.1212/WNL.0000000000207967 37816646 PMC10752640

[3] Dabi A Banerjee P Narvaez Caicedo C Rodríguez Fernández JM. Machine learning in neurocritical care: overview, pitfalls, and potential solutions. J Neurol Neurol Disord. (2024) 10(1):105.

[4] Kung TH Cheatham M Medenilla A Sillos C De Leon L Elepaño C Performance of ChatGPT on USMLE: potential for AI-assisted medical education using large language models. PLoS Digit Health. (2023) 2(2):e0000198. 10.1371/journal.pdig.0000198 36812645 PMC9931230

[5] Vinny PW Vishnu VY Srivastava MVP. Artificial intelligence shaping the future of neurology practice. Med J Armed Forces India. (2021) 77(3):276–82. 10.1016/j.mjafi.2021.06.003 34305279 PMC8282510

[6] Ali R Tang OY Connolly ID Zadnik Sullivan PL Shin JH Fridley JS Performance of ChatGPT and GPT-4 on neurosurgery written board examinations. Neurosurgery. (2023) 93(6):1353–65. 10.1227/neu.0000000000002632 37581444

[7] Figari Jordan R Sandrone S Southerland AM. Opportunities and challenges for incorporating artificial intelligence and natural language processing in neurology education. Neurology. (2024) 3(1):e200116. 10.1212/NE9.0000000000200116 39360153 PMC11441748

[8] Bhayana R Krishna S Bleakney RR. Performance of ChatGPT on radiology board-style examination: insights into current strengths and limitations. Radiology. (2023) 307(5):e230582. 10.1148/radiol.230582 37191485

[9] Gilson A Safranek CW Huang T Socrates V Chi L Taylor RA How does ChatGPT perform on the United States medical licensing examination (USMLE)? The implications of large language models for medical education and knowledge assessment. JMIR Med Educ. (2023) 9:e45312. 10.2196/45312 36753318 PMC9947764

[10] Mihalache A Popovic MM Muni RH. Performance of an artificial intelligence chatbot in ophthalmic knowledge assessment. JAMA Ophthalmol. (2023) 141(6):589–97. 10.1001/jamaophthalmol.2023.1144 37103928 PMC10141269

[11] American Academy of Neurology. Residency in-Service Training Examination (RITE) Content Outline. Minneapolis, MN: American Academy of Neurology (2024). Available online at: https://www.aan.com/tools-resources/residency-in-service-training-examination (Accessed October 21, 2025).

[12] American Board of Psychiatry and Neurology. Instructions for the Neurology Certification Examination. Deerfield, IL: American Board of Psychiatry and Neurology (2024). Available online at: https://www.abpn.com/wp-content/uploads/2020/11/2021_Neurology_CERT_Format_and_Scoring.pdf (Accessed October 21, 2025).

[13] Bloom BS Engelhart MD Furst EJ Hill WH Krathwohl DR. Taxonomy of Educational Objectives: The Classification of Educational Goals. Handbook I: Cognitive Domain. New York: David McKay Company (1956).

[14] Schubert MC Wick W Venkataramani V. Performance of large language models on a neurology board-style examination. JAMA Netw Open. (2023) 6(12):e2346721. 10.1001/jamanetworkopen.2023.46721 38060223 PMC10704278

[15] Ros-Arlanzón P Pérez-Sempere A. Evaluating AI competence in specialized medicine: comparative analysis of ChatGPT and neurologists in a neurology specialist examination in Spain. JMIR Med Educ. (2024) 10:e56762. 10.2196/56762 39622707 PMC11611784

[16] Inojosa H Ramezanzadeh A Gasparovic-Curtini I Wiest I Kather JN Gilbert S Integrating large language models in care, research, and education in multiple sclerosis management. Mult Scler. (2024) 30(11–12):1392–401. 10.1177/13524585241277376 39308156 PMC11514324

[17] Barrit S Torcida N Mazeraud A Boulogne S Benoit J Carette T Specialized large language model outperforms neurologists at complex diagnosis in blinded case-based evaluation. Brain Sci. (2025) 15(4):347. 10.3390/brainsci15040347 40309809 PMC12025783

[18] Moura L Jones DT Sheikh IS Murphy S Kalfin M Kummer BR Implications of large language models for quality and efficiency of neurologic care: emerging issues in neurology. Neurology. (2024) 102(11):e209497. 10.1212/WNL.0000000000209497 38759131

[19] Matsoukas S Morey J Lock G Chada D Shigematsu T Marayati NF AI software detection of large vessel occlusion stroke on CT angiography: a real-world prospective diagnostic accuracy study. J Neurointerv Surg. (2023) 15(1):52–6. 10.1136/neurintsurg-2021-018391 35086962

[20] Grzybowski A Brona P Lim G Ruamviboonsuk P Tan GSW Abramoff M Artificial intelligence for diabetic retinopathy screening: a review. Eye. (2020) 34(3):451–60. 10.1038/s41433-019-0566-0 31488886 PMC7055592

[21] Yuan J Li H Ding X Xie W Li YJ Zhao W Give me FP32 or give me death? Challenges and solutions for reproducible reasoning. *arXiv* [Preprint]. *arXiv:2506.09501* (2025). Available online at: https://arxiv.org/abs/2506.09501 (Accessed October 21, 2025).

[22] Wei B Boxiong B. Performance evaluation and implications of large language models in radiology board exams: prospective comparative analysis. JMIR Med Educ. (2025) 11:e64284. 10.2196/64284 39819381 PMC11756834

[23] Tam ZR Wu CK Lin CY Chen YN. None of the above, less of the right: parallel patterns in human and LLM performance on multiple-choice question answering. In: Che W Nabende J Shutova E Pilehvar MT, editors. Findings of the Association for Computational Linguistics: ACL 2025. Vienna: Association for Computational Linguistics (2025). p. 20112–34. Available online at: https://aclanthology.org/2025.findings-acl.1031 (Accessed October 21, 2025).

